# My favourite flowering image: an Arabidopsis inflorescence expressing fluorescent reporters for the *APETALA3* and *SUPERMAN* genes

**DOI:** 10.1093/jxb/ery098

**Published:** 2018-04-06

**Authors:** Nathanaël Prunet

**Affiliations:** 1 Howard Hughes Medical Institute, California Institute of Technology, Division of Biology and Biological Engineering, Pasadena, CA, USA; 2 Trinity College Dublin, Ireland

**Keywords:** Arabidopsis, flower development, flower meristem, boundary formation, floral organ identity, APETALA3, SUPERMAN

**Fig. 1.**
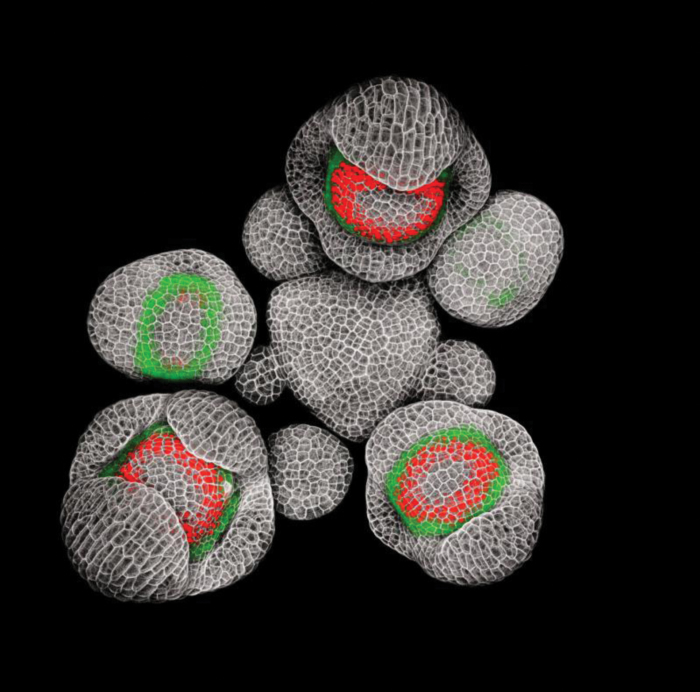
*AP3* and *SUP* expression in young Arabidopsis flower buds. Arabidopsis inflorescence expressing *gAP3-GFP* (green) and *gSUP-3xVenusN7* (red) fluorescent reporters. Cell walls were stained with propidium iodide (grey). Siliques and older flower buds were removed, and the inflorescence was prepared and imaged on a Zeiss LSM780 with a 20× water-dipping lens as described in Prunet (2017) and Prunet *et al.* (2016). Background noise was digitally removed for aesthetic reasons.

When asked to provide a picture for the cover of the Flowering Newsletter, I picked this image of an *Arabidopsis thaliana* inflorescence expressing fluorescent reporters for two key regulators of flower development: *APETALA3* (*AP3*), which promotes petal and stamen identity, and *SUPERMAN* (*SUP*), which encodes a transcriptional repressor that defines the boundary between stamens and pistil (Fig. 1). The choice was easy: it was an important breakthrough in my research on the role of *SUP* in the separation of stamens in whorl 3 and carpels in whorl 4; and among the images of flowers I have taken with a confocal microscope, it is also one my favourites aesthetically. The image won awards at the 2015 Nikon Small World and FASEB BioArt competitions and is published in Prunet *et al.* (2017).

The molecular mechanisms underlying the determination of floral organ identity have been extensively studied over the last three decades, from the description of mutants with floral organ homeosis (Bowman *et al.*, 1989, 1991; Irish and Sussex, 1990) to the characterization of the corresponding genes, most of which encode transcription factors of the MADS-box family (Yanofsky *et al.*, 1990; Jack *et al.*, 1992; Mandel *et al.*, 1992; Goto and Meyerowitz, 1994), and the identification of their targets (Kaufmann *et al.*, 2009, 2010; Wuest *et al.*, 2012; Ó’Maoiléidigh *et al.*, 2013). Floral organ identity is determined by the combinatorial action of four classes of MADS-box transcription factors [class A, AP1; class B, AP3 and PISTILLATA (PI); class C, AGAMOUS (AG); and class E, SEPALLATAs (SEPs)], which form different protein quartets in each whorl (reviewed in Prunet and Jack, 2014). For instance, quartets composed of class B, C, and E transcription factors orchestrate stamen development in whorl 3, while quartets composed of class C and E transcription factors alone determine carpel identity in whorl 4. These quartets recruit different transcription co-regulators and histone modification factors to regulate the transcription of their targets (Smaczniak *et al.*, 2012). While the genetic networks downstream of these quartets have been partially deciphered (reviewed in Stewart *et al.*, 2016), questions remain about how boundaries between floral whorls are established.

Mutations in *SUP* disrupt the boundary between whorls 3 and 4, with the formation of numerous extra stamens, usually at the expense of carpels, which are reduced or missing in most alleles (Schultz *et al.*, 1991; Bowman *et al.*, 1992). This phenotype is associated with the expansion of the expression of *AP3* and *PI* towards the center of the flower (Bowman *et al.*, 1992; Goto and Meyerowitz, 1994), but does not result from a simple homeotic conversion of carpels into stamens: the overall number of floral organs is increased in *sup* compared to the wild type, indicating an excess of cell proliferation in *sup* flowers. Two models have been proposed for the developmental origin of the extra stamens in *sup* flowers. It was first suggested that these extra stamens form in whorl 4, due to the ectopic expression of class B genes, and that the increase in floral organ number comes from delayed termination of the floral stem cells (Schultz *et al.*, 1991; Bowman *et al.*, 1992). However, when the *SUP* gene was identified, *in situ* hybridization experiments suggested that *SUP* was co-expressed with *AP3* and *PI* in the inner part of whorl 3, but not expressed in whorl 4, casting doubts on the fact that SUP might function to prevent ectopic expression of class B genes in the fourth whorl (Sakai *et al.*, 1995). Instead, SUP was proposed to control the balance of cell proliferation between whorls 3 and 4. According to this new model, extra stamens arise from whorl 3 cells that over-proliferate, while reduced proliferation in whorl 4 results in a loss of carpel tissue (Sakai *et al.*, 1995, 2000). For more than 25 years after the isolation of the *sup* mutant it had not been possible to discriminate between these two models. This was mostly due to limitations in the techniques that were used at the time, such as *in situ* hybridizations or GUS reporter lines, which lack sufficient cellular resolution and cannot not be used on live tissues. The image I have chosen helped solve this question.

I first became interested in *SUP* during my PhD with Christophe Trehin and Ioan Negrutiu at *École Normale Supérieure de Lyon*. I was studying three different mutants with a minor delay in the termination of floral stem cells that was manifesting through a slight increase in the number of carpels and the occasional formation of extra organs inside the gynoecium (Prunet *et al.*, 2008). This phenotype was correlated with a decrease in the expression of *AG*—which acts as the main switch to terminate floral stem cells (Lenhard *et al.*, 2001; Lohmann *et al.*, 2001)—in the center of the flower meristem (Prunet *et al.*, 2008). However, the combination of these three mutations resulted in a spectacular phenotype, with the formation of an indeterminate spiral of stamens at the center of the flower. This phenotype is also observed when combining the *sup-1* mutation with the moderate loss-of-function allele *ag-4* (Prunet *et al.*, 2008). While *SUP* initially appeared at the margin of the genetic networks I was studying, I started to increasingly suspect that it was involved in the timely termination of floral stem cells.

When I started my postdoc in Tom Jack’s lab at Dartmouth College, I decided to investigate the function of *SUP* using a live confocal imaging approach—a technique that allows us to monitor the expression of multiple genes in live tissue with good cellular resolution. Our data supported the model in which extra stamens in *sup* mutant flowers arise from whorl 4 rather than whorl 3. We observed a prolonged expression of the stem cell marker *CLAVATA3* and stem cell activator *WUSCHEL* in *sup* flowers compared to the wild type, suggesting that the increase in floral organ number resulted from delayed termination of the floral stem cells rather than from an over-proliferation of cells in whorl 3 (Prunet *et al.*, 2017). Time-lapse experiments also demonstrated that a ring of cells in whorl 4, adjacent to the boundary with whorl 3, starts expressing *AP3* ectopically at the transition between whorl 4 and 5 in *sup* mutant flowers, thus confirming that extra stamens form in the fourth whorl in *sup* (Prunet *et al.*, 2017). Our data also seemed to point at a mostly non cell-autonomous effect of SUP, which, based on hard-to-interpret *in situ* hybridizations, was believed to be expressed in whorl 3, and not in whorl 4 (Sakai *et al.*, 1995). We generated a translational fluorescent reporter for *SUP* to have a closer look at the *SUP* expression pattern. It turned out to be a slow and painful process—it took 4 years and some pretty acrobatic cloning by Tom—but we finally obtained a fluorescent *SUP* reporter just as I moved from Dartmouth to Elliot Meyerowitz’s lab at Caltech. This image of an Arabidopsis inflorescence expressing two translational reporters for *AP3* (fused with a single GFP) and *SUP* (fused with three Venus proteins and a nuclear localization signal) was one of the first images I took at Caltech; it was also the first time I managed to separate signals from GFP and YFP, which have partially overlapping emission spectra. But most importantly, this image clearly showed that contrary to what was previously thought, *SUP* is expressed on both sides of the boundary between whorls 3 and 4, not just in whorl 3. *SUP* and *AP3* are expressed along two opposite gradients that only partially overlap in whorl 3, and whorl 4 cells that express *SUP* in wild-type flowers at stage 5 ectopically express *AP3* instead in *sup* mutant flowers, indicating that *SUP* prevents *AP3* expression in whorl 4 in a cell-autonomous manner (Prunet *et al.*, 2017).

Independently of the scientific significance of this image, I love it for aesthetic reasons. One of the reasons why I studied biology in the first place is that of all sciences, it leaves the most room for artistic expression: observational drawing is an integral part of the learning process. This science-meets-art aspect—for which the term SciArt has been coined—has long been an important driver for my work. I chose to study development for my PhD because of the rich imaging possibilities this field offers. I later based my postdoc research on a confocal imaging approach for the power of the technique to solve developmental questions but also for the beauty of the images that can be generated. I admit to spending more time on the microscope than strictly required to answer my initial scientific questions, trying to get aesthetically perfect images (and I consider myself lucky to work with Elliot, who has been very supportive of that). But then, as Samuel H. Scudder noticed once he decided to draw his fish (‘At last a happy thought struck me—I would draw the fish; and now with surprise I began to discover new features in the creature. Just then the Professor returned. ‘That is right’, said he; ‘a pencil is one of the best of eyes’; Scudder, 1974), carefully crafted images often bring to our attention interesting biological details that we would not have suspected otherwise.
